# Retrospective In-Hospital Mortality Analysis of GeriatricPatients Treated in a Level 1 Trauma Center

**DOI:** 10.3390/jcm12103466

**Published:** 2023-05-15

**Authors:** Sebastian Höller, Lina F. Wübbeke, Jamina Apel, Thelonius Hawellek, Stephan Sehmisch, John Wiedenhöft, Wolfgang Lehmann, Daniel B. Hoffmann

**Affiliations:** 1 Department of Trauma, Orthopedic and Plastic Surgery, University Medical Center Göttingen (UMG), 37075 Göttingen, Germany; 2 Department of Trauma Surgery, Medical School Hannover, 30625 Hannover, Germany; 3 Scientific Core Facility for Medical Biometry and Statistical Bioinformatics (MBSB), University Medical Center Göttingen (UMG), 37075 Göttingen, Germany

**Keywords:** geriatric, mortality, surgery

## Abstract

The aim of this study is to determine the critical time intervals and influencing covariates for in-hospital mortality in geriatric trauma and orthopedic patients. During a period of five years, we retrospectively review patients aged > 60 years who were hospitalized at the Department of Trauma, Orthopedic, and Plastic Surgery. The primary outcome is the mean time to death. Survival analysis is performed using an accelerated failure time model. A total of 5388 patients are included in the analysis. Two-thirds underwent surgery (n = 3497, 65%) and one-third were conservatively treated (n = 1891, 35%). The in-hospital mortality rate is 3.1% (n = 168; surgery, n = 112; conservative, n = 56). The mean time to death is 23.3 days (±18.8) after admission in the surgery group and 11.3 days (±12.5) in the conservative treatment group. The greatest accelerating effect on mortality is found in the intensive care unit (16.52, *p* < 0.001). We are able to identify a critical time interval for in-hospital mortality between days 11 and 23. The day of death on weekend days/holidays, hospitalization for conservative treatment, and treatment at the intensive care unit significantly increase the risk of in-hospital mortality. Early mobilization and a short hospitalization duration seem to be of major importance in fragile patients.

## 1. Introduction

There is an increasing number of geriatric patients treated at a department of trauma and orthopedic surgery because of the overall aging population. In 2018, 101.1 million people in the European Union were older than 65 years. This number is expected to increase up to 149.2 million in 2050, a relative increase from 19% to 28.5% [1]. In the United States, the number of people aged older than 65 years is even expected to have more than doubled by 2060 [2].

Geriatric patients are at an increased risk of trauma because of deficits in the visual, auditory, nervous, and musculoskeletal systems. Slowing reaction time, reduced visual acuity and consciousness, reduced muscle tone, and postural instability lead to a high incidence of falls and subsequent injuries [3,4]. In addition, comorbidities and the need of drug intake can lead to an increased fall tendency. Despite increased fall awareness and prevention strategies, there are still high visiting rates in emergency departments for fall-related injuries among patients older than 65 years [5].

Osteoporosis becomes more likely with increasing age, leading to reduced bone mass and strength and an increased risk of fractures. In addition, drug intake that is due to comorbidities can influence bone mass as well. The so-called fragility fractures follow a low-stress impact that does not break a healthy bone [6]. Osteoporosis causes more than 9 million fragility fractures annually worldwide [7]. The current report of the International Osteoporosis Foundation (IOF) predicts a 23% increase in fragility fractures in the largest countries of the European Union until 2030. The increasing number of geriatric patients and the increasing fracture frequency among the elderly is a challenge to healthcare systems worldwide owing to increased costs. The IOF expect a 27% increase in fracture-related costs until 2030 [6,8].

A recent nationwide epidemiological study analyzed the incidence of different fracture types in Germany from 2009 to 2019. The authors reported an overall increase of 14% in the last decade. The most common fracture types are hip and distal radius fractures [8]. Hip fractures are not only the most common fall-related injury but also cause surgery in geriatric patients and are associated with a high one-year mortality rate of up to 30% [3,9,10]. The current literature often focuses on mortality after hip fracture and hip replacement surgery [9,10,11,12].

Epidemiological data from Germany in 2019 showed that other fracture types have substantial incidences [8]. Mortality after treatment for other fracture types or fracture-independent diseases in patients in the special field of trauma and orthopedic surgery has been assessed in a few studies. However, this is important because degenerative diseases of the musculoskeletal system are more common with increasing age. Hip and knee replacements are common planned surgical procedures [13,14]. Additionally, the incidence of degenerative spinal disorders leading to surgical treatment is increasing among older patients [15].

Advanced age is a well-known risk factor for poor outcomes of mortality and morbidity after any kind of surgical procedure [16]. Increased multimorbidity makes the treatment of geriatric patients challenging for orthopedic and traumatic surgeons. Much effort has been made to study mid-term and long-term mortality rates, but less is known about perioperative mortality during the hospital stay.

The aim of the present study was to determine the critical time intervals for in-hospital mortality in geriatric patients treated at a department of trauma and orthopedic surgery. Additionally, the influence of different covariates on in-hospital mortality was examined.

## 2. Materials and Methods

### 2.1. Study Design, Setting, Participants

Between 2013 and 2017, we retrospectively analyzed mortality in patients aged > 60 years at the Department of Trauma Surgery, Orthopaedics, and Plastic Surgery of the University Medical Center Göttingen, a level 1 trauma center in Germany. The local ethics committee approved this study. All the research was performed in accordance with the principles of the Declaration of Helsinki. The Strengthening the Reporting of Observational Studies in Epidemiology (STROBE) guidelines for cohort studies were followed.

All patients treated in the Department of Trauma, Orthopedic, and Plastic Surgery were retrospectively reviewed. Patients younger than 60 years and those who died within 24 h after admission were excluded. All patients older than 60 years who died during their hospital stay were included. Patients were divided into two groups based on the type of treatment received (surgery vs. conservative treatment) and further analyzed.

### 2.2. Data Collection

All data were retrospectively collected using medical files and medical reports, death certificates, and the results of blood and microbiological sample analyses. Baseline characteristics (age, sex), presence of resistant bacterial strains, type of admission, day of admission and discharge (workday, weekend/holiday), length of hospital stay, and ward at the time of discharge or death (normal ward, intermediate care unit, intensive care unit, or palliative care unit) were collected for all patients. Additional comorbidities (Charlson comorbidity score, body mass index, anticoagulative medication, and multi-resistant bacterial strains), the reason for inpatient admission (diagnosis at admission), and type of operation for surgical-treated patients were recorded for patients who died during the hospital stay. Finally, the time of death and cause of death were documented for patients who died.

Multi-resistant bacterial strains were defined as so-called ESKAPE pathogens (the Enterococcus faecium, Staphylococcus aureus, Klebsiella pneumoniae, Acinetobacter baumannii, Pseudomonas aeruginosa, and Enterobacter species) [17]. The Charlson comorbidity score is a scoring system that predicts the mortality rate depending on the comorbidities of the patient. Nineteen comorbidities contributed to the calculation of the index with different weightings. In addition, the patient’s age was added [18].

### 2.3. Study Groups and Endpoints

Patients were divided into two groups based on the type of treatment received: surgery or conservative treatment. The primary endpoint for both groups was the mean time until death (in days). The reason for death and the ward at the time of death were analyzed as secondary outcomes.

### 2.4. Statistical Methods

All statistical analyses were performed in R (version 3.6.0, the R foundation for statistical computing, Vienna, Austria) using the survival package (version 3.2-7). Standard descriptive statistics were used for demographic data and baseline characteristics. Categorical data are expressed as counts and percentages. Continuous data are presented as the mean and standard deviation. Survival analysis was initially performed using the standard Cox proportional hazard model; however, while a nonsignificant χ^2^ test for the Schoenfeld residuals supported the proportional hazards assumption, the Cox–Snell residuals exhibited a clear nonlinear trend. Instead, an accelerated failure time model (AFT) was used, though results qualitatively matched those of the Cox model. The error distribution was chosen by minimizing the Akaike information criterion (AIC), with the log-logistic distribution yielding the best value. The Kaplan–Meier plot of the AFT residuals against the log-logistic distribution confirmed the fit of the model. Statistical significance was set at *p* < 0.05.

## 3. Results

**Figure 1:**
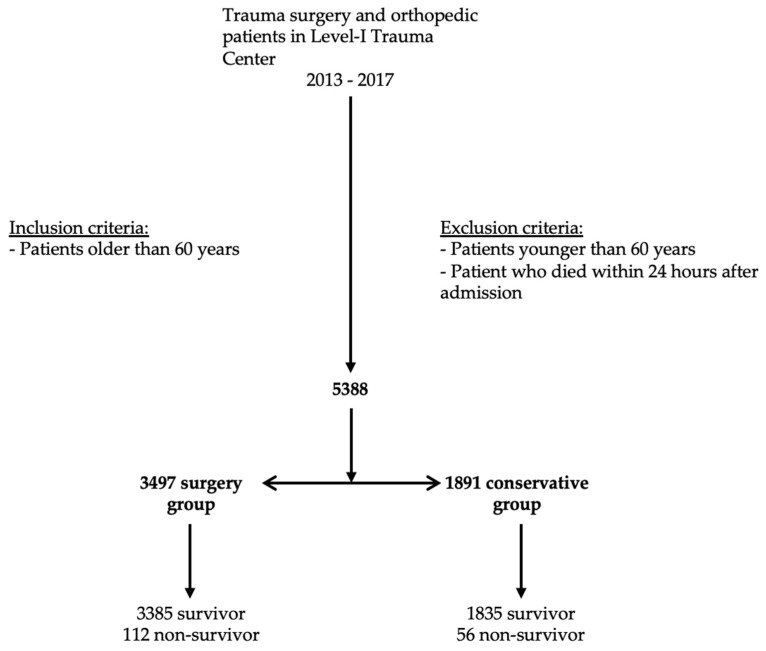
Flow chart.

During the study period from 2013 to 2017, 5388 patients older than 60 years were treated at the Department of Orthopedic and Trauma Surgery. Two-thirds of these patients underwent surgical treatment (3497 patients, 65%) compared to one-third of the conservatively treated patients (1891 patients, 35%). A total of 168 patients died during the hospital stay (in-hospital mortality rate, 3.1%). According to the two treatment groups, 112 patients who died underwent surgical treatment and 56 patients underwent conservative treatment (Figure 1).

After the recruitment process, the baseline characteristics of both groups were analyzed (Table 1). The survivor group showed a mean length of hospital stay of 13.5 days (±13.1) and non-survivors of 23.3 days (±18.8) after surgery. The survivors in the conservatively treated and surgical group had a mean length of hospital stay of 4.4 days (±4.3) and 11.3 days (±12.5), respectively. Most patients in all groups were admitted and discharged on workdays. Resistant bacterial strains were unevenly distributed among the groups. In the surgically treated group, resistant bacterial strains were found in 58.9% of the survivors and 3.1% of the non-survivors. In the conservatively treated group, resistant bacterial strains were found in 26.8% of survivors and 1.4% of non-survivors.

Additional baseline characteristics were collected for 168 patients who died during their hospital stay (Table 2). The mean age was older than 80 years in both groups (surgery: 80.3 years ± 8.9; conservative: 81.1 years ± 8.7). Sex was evenly distributed in both the groups. Regarding comorbidities, the Charlson Comorbidity Index was 2.18 points in the surgery group and 2.34 in the conservative treatment group. In both groups, almost half of the patients were on anticoagulants. In the surgically treated group, a multidrug-resistant bacterial strain was found in 28% of patients. Most surgically treated patients were admitted and treated for fractures of the hip and pelvis. In relation to this, most surgically treated patients receive osteosynthesis or prosthesis implantation. The second largest group comprised patients with infections (20.5%), with or without underlying osteosynthesis material, treated with debridement and removal of the foreign material, if needed. In the conservatively treated group, conservative treatment of the pelvis, vertebrae, or other fractures was the reason for admission in approximately 10–15%, respectively. However, most patients without surgery were admitted because of immobilization of back pain, concussion, or contusions aiming for proper analgesia and mobilization during the hospital stay (58.9%).

### Mortality Analysis

The mean time until death was 23.3 days (±18.8) after admission in the surgery group and 11.3 days (±12.5) in the conservative treatment group (Table 3). In the surgery group, the most common ward at the time of death was the intensive care unit (59 cases, 52.7%), followed by the intermediate care unit (23 cases, 20.5%), the normal ward (20 cases, 17.9%), and the palliative care unit (10 cases, 8.9%). After conservative treatment, 20 patients (35.7%) died in the intensive care unit, 16 (28.6%) in a normal ward, 13 (23.2%) in an intermediate care unit, and 7 (12.5%) in a palliative care unit. Heart failure (27 patients, 24.1%) and sepsis (25 patients, 22.3%) were the two main causes of death in the surgery group. Less common respiratory failure (17 patients, 15.2%) led to death in surgically treated patients. Conservatively treated patients died mainly because of respiratory failure (15 cases, 26.8%), sepsis (11 cases, 19.6%), and heart failure (10 cases, 17.9%). The causes of death are listed in Table 3.

An AFT model was used to perform survival analysis and identify covariates with significant effects on patient mortality (Table 4). Except for sex, type of admission, and day of admission, all other factors showed a significant accelerating effect on mortality. Increasing age and the presence of resistant bacterial strains had the smallest acceleration effect (1.05 (*p* < 0.001) and 1.50 (*p* = 0.018), respectively). The day of discharge/death showed an acceleration factor of 2.29 for discharge or death on weekends and public holidays compared to workdays (*p* < 0.001). Conservative treatment also led to a significantly faster progression (2.88, *p* < 0.001). The greatest acceleration factor was found in the intensive care unit compared to the normal ward (18.23; *p* < 0.001). Both the intermediate and palliative care units had a significant accelerating effect compared to the normal ward (intermediate care 2.52, palliative 9.06; *p* < 0.001).

## 4. Discussion

The increasing number of geriatric patients with increased multimorbidity treated at departments of trauma and orthopedic surgery is challenging for responsible surgeons. Advanced age is a risk factor for poor outcomes regarding mortality after any type of surgery [16]. Much effort has been made to study mid-term and long-term mortality rates, but less is known about the critical time intervals for in-hospital mortality. However, this is of major importance regarding the possibility of prevention.

In our study, we analyzed in-hospital mortality in a large cohort of more than 5300 patients older than 60 years to determine these critical time intervals and to identify covariates that influence in-hospital mortality. An overall in-hospital mortality rate of 3.1% was found in this study, which is comparable to the results in the existing literature. Zerah et al. recently reported an in-hospital mortality rate of 3.2% in 1000 patients older > 70 years with hip fractures [11]. Bliemel et al. reported an in-hospital mortality rate of 6% in a prospective study of patients hospitalized for the same reason [12].

As expected from a department of orthopedic and trauma surgery, two-thirds of the patients underwent surgical treatment. The mean length of hospital stay was 12.8 days after surgery and 4.8 days after conservative treatment, respectively. Bugaevsky et al. reported a hospitalization length of 8.3–12.1 days in patients treated surgically for hip fractures [9]. Bliemel et al. reported a mean length of hospital stay of 14 days in a cohort of geriatric hip fracture patients [12]. Data on hospitalized, conservatively treated geriatric patients are rare. Ernstberger et al. compared conservatively and surgically treated geriatric patients with acetabulum fractures and found a hospitalization length of 12.9 days in conservatively treated patients [19].

In our conservatively treated group of patients, most admissions were emergency, as expected. Only a small subgroup was labeled as planned admission. These patients suffered mainly from immobilization caused by back pain or soft tissue infection. Outpatient treatment with oral analgesia or antibiotics was initiated but not successful; therefore, inpatient treatment was followed.

We found a difference of 12 days in the mean time until death between the surgery group (23.3 days) and the conservatively treated group (11.3 days). Therefore, based on our results, we have a critical time window for in-hospital mortality between days 11 and 23. Bugaevsky et al. performed a retrospective cohort study including 441 patients older than 65 years who underwent surgery for hip fractures, analyzing differences in outcomes between treatment at an orthogeriatric unit (OG-unit) versus an orthopedic department (OD). The mean duration of hospitalization at the OG-unit was 12.1 days, while that at the OD 8.3 days [9]. This example shows that most patients after hip fracture surgery have already been discharged before the critical time window begins. The survivors after surgical treatment had a mean length of hospital stay of 12.8 days as well. Therefore, early mobilization and a short hospitalization duration are of major importance for these fragile patients. Respiratory failure and pulmonary artery embolism were the two most common causes of death in our study. It was even the leading cause of death during conservative treatment. These two causes of death can be related to immobilization during a long hospital stay and can be prevented by early mobilization and early discharge for rehabilitation to reduce the risk of respiratory failure and thrombosis. In a recent population-based study by Chen et al., patients who developed a pulmonary artery embolism perioperatively had a longer length of hospital stay as well [20].

The days of admission and discharge or death, divided into workdays or weekend days and holidays, were collected to investigate the effect of possibly reducing capacities and quality of patient care. Our AFT model revealed days of discharge/death as a moderate acceleration factor (2.24 for mortality compared with workdays). A comparable accelerating effect was associated with conservative treatment. The greatest acceleration factor was found for the intensive care unit compared to the normal ward, with a factor of 16.52. This is not surprising, since patients only go to the intensive care unit because of severe diseases, and very old intensive care patients are known to have a poor survival rate. A European-wide observational study including 5000 very elderly patients treated at an intensive care unit showed a mortality rate of 22.1% during treatment in the intensive care unit [21].

The term “frailty” as geriatric syndrome, characterized by an increased vulnerability that is due to an age-related decline in multiple physiological systems, has become a subject of increasing interest [2,16]. A systematic review assessed the relationship between frailty and postoperative outcomes in geriatric patients in 23 studies and revealed strong evidence that frailty can predict postoperative mortality and a prolonged length of hospital stay. The authors concluded that frailty assessment may be a valuable tool for perioperative assessment [16]. Notably, 21 different methods were used to measure frailty in 23 studies. Standardized validated frailty instruments are needed for implementation in clinical practice [2].

### Limitations

A limitation of the present study that must be addressed is its retrospective nature. All possible disadvantages of this design, such as recall bias and missing data, may exist. Although this study is one of the largest available series, the opportunities for data collection were limited owing to its retrospective design. The baseline characteristics of the patients were limited. The group sizes were unevenly distributed. In addition, we had to face a very heterogeneous group of patients since we included all patients treated during the study period. On the one hand, surgery was performed because of a hip fracture; in contrast, the patient was hospitalized for pain medication and mobilization after contusion because of immobilizing back pain. This heterogeneous group is an advantage on the one hand, since it reflects the real average group of patients every department of trauma and orthopedic surgery is facing. However, this may have led to confounding bias, and many covariates were not examined.

## 5. Conclusions

This study presents a large cohort of more than 5300 patients older than 60 years who were treated at a department of trauma and orthopedic surgery. We were able to identify a critical time interval for in-hospital mortality between days 11 and 23. The day of death on weekend days/holidays, hospitalization for conservative treatment, and treatment at the intensive care unit significantly increased the risk of in-hospital mortality. Respiratory failure and pulmonary artery embolism were the two leading causes of death in our study, particularly in conservatively treated patients. Therefore, early mobilization and a short hospitalization duration are of major importance in these fragile patients.

## Figures and Tables

**Table 1:** Baseline characteristics of all patients categorized by surgical and conservative treatment.

	Surgery		Conservative	
	Survivor (n = 3385)	Non-Survivor (n = 112)	Survivor (n = 1835)	Non-Survivor (n = 56)
Age, years				
Mean (SD)	73.7 (±8.7)	80.3 (±8.9)	77.9 (±9.4)	81.1 (±8.7)
Median (Range)	73.0 (60, 101)	81.5 (61, 98)	78.0 (61, 103)	81.5 (64, 99)
Gender				
Male	1591 (41.2%)	60 (53.6%)	899 (41.4%)	24 (42.9%)
Female	2271 (58.8%)	52 (46.4%)	1120 (58.6%)	32 (57.1%)
Length of hospital stay, days				
Mean (SD)	13.5 (±13.1)	23.3 (±18.8)	4.4 (±4.3)	11.3 (±12.5)
Type of admission				
Planned	1864 (48.3%)	9 (8.0%)	160 (7.4%)	2 (3.6%)
Emergency	1998 (51.7%)	103 (92.0%)	2011 (92.6%)	54 (96.4%)
Resistant bacterial strain	119 (3.1%)	66 (58.9%)	30 (1.4%)	15 (26.8%)
Day of admission				
Workday	3328 (86.2%)	94 (83.9%)	1603 (73.8%)	43 (76.8%)
Weekend/Holiday	534 (13.8%)	18 (16.1%)	568 (26.2%)	13 (23.2%)
Day of discharge or death				
Workday	3425 (88.7%)	70 (62.5%)	1881 (86.6%)	41 (73.2%)
Weekend/Holiday	437 (11.3%)	42 (37.5%)	290 (13.4%)	15 (26.8%)
Ward at time of discharge or death				
Normal ward	3656 (94.7%)	20 (17.9%)	1988 (91.6%)	16 (28.6%)
Intermediate care unit	203 (5.3%)	23 (20.5%)	182 (8.4%)	13 (23.2%)
Intensive care unit	3 (0.1%)	59 (52.7%)	1 (0.0%)	20 (35.7%)
Palliative care unit	0 (0%)	10 (8.9%)	0 (0%)	7 (12.5%)

Categorical data are expressed as counts and percentages, and continuous data as mean + standard deviation.

**Table 2:** Additional baseline characteristics of patients who died during hospital stay (n = 168).

	Surgery (n = 112)	Conservative (n = 56)
Age, years, mean (SD)	80.3 (±8.9)	81.1 (±8.7)
Gender		
Male	60 (53.6%)	24 (42.9%)
Female	52 (46.4%)	32 (57.1%)
Charlson Comorbidity Index	2.18	2.34
Body Mass Index, kg/m^2^, mean (SD)	24.4 (±SD)	26.8 (±SD)
Anticoagulation	46 (41.1%)	25 (44.6%)
Multi-resistant bacterial strain	31 (27.7%)	3 (5.4%)
Diagnosis at admission		
Fracture of hip/pelvis	44 (39.3%)	8 (14.3%)
Vertebral fractures	12 (10.7%)	7 (12.5%)
Other fractures	13 (11.6%)	6 (10.7%)
Discitis	7 (6.3%)	-
Other infections	23 (20.5%)	2 (3.6%)
Other (soft tissue injuries, hematoma)	13 (11.6%)	-
Other (back pain, concussion, contusions)	-	33 (58.9%)
Kind of operation		
Osteosynthesis	61	
Prosthesis implantation	22	
Soft tissue surgery	8	
Removal osteosynthesis material	6	
Prosthesis explant	4	
Amputation	6	
Debridement	5	
Other	9	

Categorical data are expressed as counts and percentages, and continuous data as mean + standard deviation.

**Table 3:** Mortality-related outcomes for patients who died during hospital stay (n = 168).

	Surgery (n = 112)	Conservative (n = 56)
Mean time until death, days (SD)	23.3 (±18.8)	11.3 (±12.5)
Ward at time of death		
Normal ward	20 (17.9%)	16 (28.6%)
Intermediate care unit	23 (20.5%)	13 (23.2%)
Intensive care unit	59 (52.7%)	20 (35.7%)
Palliative care unit	10 (8.9%)	7 (12.5%)
Cause of death		
Heart failure	27 (24.1%)	10 (17.9%)
Sepsis	25 (22.3%)	11 (19.6%)
Respiratory failure	17 (15.2%)	15 (26.8%)
Kidney failure	10 (8.9%)	3 (5.4%)
Pulmonary artery embolism	4 (3.6%)	4 (7.1%)
Multiorgan failure	8 (7.1%)	4 (7.1%)
Other	21 (18.8%)	9 (16.1%)

Categorical data are expressed as counts and percentages, and continuous data as mean + standard deviation.

**Table 4:** Accelerated failure time model.

Variable	Coefficient	Standard Error	*z*-Value	*p*-Value	Significance	Survival Time Factor	Acceleration Factor
Intercept	10.1801	0.78	12.99	0.0000	***	26,373.56	0.00
Age	−0.0458	0.01	−5.20	0.0000	***	0.96	1.05
Gender: woman	−0.0014	0.14	−0.01	0.9923		1.00	1.00
Admission: emergency	−0.4860	0.26	−1.84	0.0656		0.62	1.63
Treatment: conservative	−1.0580	0.15	−7.00	0.0000	***	0.35	2.88
Resistant bacterial strain	−0.4069	0.17	−2.36	0.0183	*	0.67	1.50
Admission day: Weekend	0.0221	0.17	0.13	0.8994		1.02	0.98
Day of discharge: Weekend	−0.8301	0.17	−5.01	0.0000	***	0.44	2.29
Ward: IMC	−0.9230	0.19	−4.83	0.0000	***	0.40	2.52
Ward: ITS	−2.9033	0.24	−12.26	0.0000	***	0.05	18.23
Ward: Palliative	−2.2036	0.32	−6.80	0.0000	***	0.11	9.06
Log (scale)	−0.4138	0.06	−6.81	0.0000	***	0.66	1.51

ITS = intensive care unit. IMC = intermediate care unit. * for *p* < 0.05, *** *p* < 0.001

## Data Availability

Not applicable.
